# Discriminating insulin resistance in middle-aged nondiabetic women using machine learning approaches

**DOI:** 10.3934/publichealth.2024034

**Published:** 2024-05-09

**Authors:** Zailing Xing, Henian Chen, Amy C. Alman

**Affiliations:** College of Public Health, University of South Florida, 13201 Bruce B. Downs Blvd, MDC 56, Tampa, FL 33612, USA

**Keywords:** insulin resistance, machine learning, women, XGBoosting, HOMA-IR

## Abstract

**Objective:**

We employed machine learning algorithms to discriminate insulin resistance (IR) in middle-aged nondiabetic women.

**Methods:**

The data was from the National Health and Nutrition Examination Survey (2007–2018). The study subjects were 2084 nondiabetic women aged 45–64. The analysis included 48 predictors. We randomly divided the data into training (n = 1667) and testing (n = 417) datasets. Four machine learning techniques were employed to discriminate IR: extreme gradient boosting (XGBoosting), random forest (RF), gradient boosting machine (GBM), and decision tree (DT). The area under the curve (AUC) of receiver operating characteristic (ROC), accuracy, sensitivity, specificity, positive predictive value, negative predictive value, and F1 score were compared as performance metrics to select the optimal technique.

**Results:**

The XGBoosting algorithm achieved a relatively high AUC of 0.93 in the training dataset and 0.86 in the testing dataset to discriminate IR using 48 predictors and was followed by the RF, GBM, and DT models. After selecting the top five predictors to build models, the XGBoost algorithm with the AUC of 0.90 (training dataset) and 0.86 (testing dataset) remained the optimal prediction model. The SHapley Additive exPlanations (SHAP) values revealed the associations between the five predictors and IR, namely BMI (strongly positive impact on IR), fasting glucose (strongly positive), HDL-C (medium negative), triglycerides (medium positive), and glycohemoglobin (medium positive). The threshold values for identifying IR were 29 kg/m^2^, 100 mg/dL, 54.5 mg/dL, 89 mg/dL, and 5.6% for BMI, glucose, HDL-C, triglycerides, and glycohemoglobin, respectively.

**Conclusion:**

The XGBoosting algorithm demonstrated superior performance metrics for discriminating IR in middle-aged nondiabetic women, with BMI, glucose, HDL-C, glycohemoglobin, and triglycerides as the top five predictors.

## 1. Introduction

Insulin resistance (IR) is a physiological state characterized by diminished responsiveness to insulin signaling in multiple tissues, including skeletal muscles, adipose tissues, and the liver [1]. This state necessitates increasing insulin secretion to maintain normal blood glucose levels [2],[3]. It is a prevalent underlying cause of metabolic syndrome, characterized by abdominal obesity, hyperlipidemia, hyperglycemia, and hypertension [4]. IR has been identified as a potential indicator for the early identification of metabolic syndrome, type 2 diabetes, and cardiovascular illnesses [5],[6]. So, identifying IR is important in safeguarding individuals' long-term health.

IR can be directly measured using a hyperinsulinemic-euglycemic clamp, but this method has drawbacks such as invasiveness, subject discomfort, and technical challenges [7]. The homeostasis model assessment of insulin resistance (HOMA-IR) is a widely used indicator for indirectly measuring IR [8]. However, fasting insulin is not typically included in routine blood tests, which could impede the identification of IR when insulin is employed to calculate HOMA-IR [9]. Straightforward determination of IR is still needed for regular screening. In addition, using machine learning techniques for disease detection and prediction has experienced a recent surge in popularity [10]. This application holds promise in enhancing our understanding of how features are associated with health conditions [11]. Although some characteristics contributing to IR have been identified [12]–[14], there is currently a lack of an appropriate model to predict IR accurately, specifically in women.

Middle-aged women are generally at a higher risk of developing IR due to age-related changes in metabolism and hormonal fluctuations [15]. The menopausal transition is associated with hormonal changes impacting insulin sensitivity [16]. Identifying and managing IR during the pivotal stage of middle age can help mitigate the risk of metabolic disorders and cardiovascular disease in later life [17]. Although some researchers attempted to predict IR using machine learning methods, their models did not account for reproductive health variables [12],[13]. Specifically targeting the female population can allow for the addition of reproductive health factors into models. Developing predictive models tailored for middle-aged women can improve risk assessment and personalized medicines and address potential disparities [18].

Therefore, we aimed to use machine learning algorithms to identify the optimal IR prediction model from demographic and behavioral factors, laboratory variables, daily nutrient intake, and reproductive health variables in middle-aged, nondiabetic American women based on the National Health and Nutrition Examination Survey (NHANES). We hypothesized that the predictors identified by machine learning methods would provide a more comprehensive metabolic health evaluation than HOMA-IR alone. Additionally, if the IR prediction model is accessible, we can use it to identify women with IR despite the absence of HOMA-IR values. By incorporating multiple biomarkers and clinical indicators, a more nuanced and holistic understanding of IR can be acquired, resulting in focused interventions and preventive strategies.

## 2. Materials and methods

### 2.1. Data source

Data was obtained from the NHANES from 2007 to 2018. The NHANES program is a cross-sectional and periodic health-related initiative in the United States administered by the National Center for Health Statistics (NCHS) of the Centers for Disease Control and Prevention. The ongoing survey and exam assess community-dwelling individuals' health and nutritional status using anthropometric measurements, health and nutrition questionnaires, and laboratory tests. The data is freely accessible to the public. The NCHS research ethics review board approved the NHANES protocol, and each participant signed informed consent forms. To obtain further details regarding the ethical approval of this research, please access: https://www.cdc.gov/nchs/nhanes/irba98.htm.

We analyzed middle-aged female NHANES participants from the 2007–2018 survey cycles that included the same variables of interest. This study defines the middle age as 45–64 years old [19]. Participants were excluded from analyses if they were male, younger than 45 years old or older than 64 years old, lacked lab testing data such as glucose, insulin, and triglycerides, or did not have information on daily nutrient intake. We also excluded individuals with diabetes mellitus or cancer, as previous research has indicated that these two health conditions could affect IR [20]. Consequently, the ultimate sample size was 2084.

### 2.2. Insulin resistance

IR was evaluated using the homeostasis model assessment of IR (HOMA-IR), which is widely employed as the predominant approach for determining IR using the formula: fasting insulin (µU/mL) × fasting glucose (mg/dL)/405 [21]. A HOMA-IR value exceeding 2.73 has previously been shown to indicate the presence of IR in nondiabetic American adults [22]. Therefore, we set the IR cutoff value for nondiabetic middle-aged women at HOMA-IR values greater than 2.73.

### 2.3. Predictors

The predictors included demographic and behavioral factors, laboratory data, daily nutritional intake, and reproductive health variables. Demographic and behavioral factors encompassed age, race (non-Hispanic white, non-Hispanic Black, Hispanic, and others), education (high school or below, and college or above), marital status (married/living with a partner, widowed/divorced/separated, and never married), family monthly poverty level index, smoking (current, former, and never), family history of diabetes, body mass index (BMI, kg/m^2^), physical activity, hypertension, systolic and diastolic blood pressure (mmHg). We defined hypertension based on the responses to the questions “Has a physician ever informed you that you have high blood pressure?” and “Are you currently taking medication for hypertension?”, systolic blood pressure ≥140 mmHg, or diastolic blood pressure ≥90 mmHg. Using metabolic equivalent scores, we measured physical activity [23].

Laboratory data included fasting glucose (mg/dL), high-density lipoprotein cholesterol (HDL-C) (mg/dL), triglyceride (mg/dL), glycohemoglobin (%), total cholesterol (mg/dL), and estimated glomerular filtration rate (eGFR, mL/min/1.73m^2^). The laboratory data-gathering procedures and tests have been documented in earlier publications [24]. We used the chronic kidney disease epidemiology creatinine equation to estimate eGFR [25].

We gathered the following information regarding daily nutritional intake: energy intake (kcal/kg), protein intake ratio (%), carbohydrate intake ratio (%), total fat intake ratio (%), total sugars (gm), dietary fiber (gm), cholesterol intake (mg), folate intake (mcg), total saturated and monounsaturated fatty acid (gm), alcohol (gm), vitamin C (mg), vitamin D (mcg), vitamin B6 (mg), vitamin B12 (mcg), caffeine (mg), iron (mg), calcium (mg), zinc (mg), sodium (mg), phosphorus (mg), magnesium (mg), copper (mg), selenium (mcg), potassium (mg), and theobromine (mg).

Reproductive variables included age at menarche, number of pregnancies, number of abortions/miscarriages/stillbirths, birth control pills, hysterectomy, bilateral oophorectomy, and female hormones. Birth control pills, hysterectomy, bilateral oophorectomy, and female hormones were categorical variables (yes and no), while others were continuous.

### 2.4. Model building process

Regarding the model construction procedure, the study subjects were first randomly separated into the training (80%) and testing (20%) datasets [26]. Next, one-hot encoding was used to encode categorical variables [27], and min-max scaling was used to standardize continuous variables [28]. This allowed the variable values to be compared across dimensions by rescaling them from 0 to 1. We utilized the Synthetic Minority Oversampling Technique in the training dataset to overcome the issue of unbalanced data. Creating synthetic samples of the minority class is a helpful approach, which improves its representation and boosts the model's ability to learn from data [29].

To examine the interrelationships among the predictors, we performed Spearman correlation analyses. If the correlation coefficient was greater than 0.75, one of two variables was removed to ensure the robustness of the model [30]. Among the 56 predictors, the following pairings of variables had correlation coefficients greater than 0.75: magnesium and potassium (0.81), folate and iron (0.76), monounsaturated fatty acid and saturated fatty acids (0.81), monounsaturated fatty acids and fat (0.84), carbohydrate and energy (0.87), energy and fat (0.86), saturated fatty acids and fat (0.77), total cholesterol and low-density lipoprotein cholesterol (0.89), BMI and waist (0.87), and number of pregnancies and number of live births (0.87). We eliminated the following variables: magnesium, folate, monounsaturated fatty acids, carbohydrate, fat, low-density lipoprotein cholesterol, waist, and number of live births, based on previous pertinent studies [12],[13].

In this study, four machine learning techniques, namely random forest (RF), extreme gradient boosting (XGBoosting), gradient boosting machine (GBM), and decision tree (DT), were chosen for hyperparameter optimization within the dataset. The RF algorithm is a type of ensemble learning that builds many decision trees during training and finds the average prediction (regression) of the individual trees or the mode of the classes (classification) [31]. XGBoosting is a distributed gradient boosting library optimized for flexible and effective implementation. Due to its speed and performance, it is scalable, highly efficient, and extensively utilized in machine learning competitions and industry applications [32]. Sequentially constructing an ensemble of weak learners (typically decision trees), GBM is renowned for its exceptional predictive accuracy and resistance to overfitting [33]. DT is a nonparametric algorithm for supervised learning in classification and regression tasks. The algorithm divides the data into subsets according to the feature values and builds a tree-like structure for making predictions [34].

Additionally, a 5-fold cross-validation method was employed. The training dataset was divided into five groups for the 5-fold cross-validation, with one group as the internal validation set and four as the internal training dataset. The average performance was computed using a grid search, and the hyperparameters were optimized to maximize the AUC of receiver operating characteristic (ROC) for the internal validation set [35]. Following the completion of model training, we utilized the testing dataset for validation.

We compared the performance metrics of different models using the AUC of ROC, accuracy, sensitivity, specificity, positive predictive value (PPV), negative predictive value (NPV), and F1 score [36]. The F1 score is a metric to quantify accuracy by precision and sensitivity [37]. Finally, we used the SHapley Additive exPlanations (SHAP) framework to illustrate the predictors' contributions and identify the threshold values of the predictors [38].

### 2.5. Statistical analysis

Since NHANES is a multistage, complicated probability sampling design, we used weighted mean (95% confidence interval) to describe continuous predictors and frequency (weighted percentage) to characterize categorical ones. We compared predictor differences between IR statuses using the Rao-Scott chi-square test for categorical variables and the t-test for continuous variables.

As participants lacking lab testing or daily nutrient intake data were excluded, other variables, except for family monthly poverty level index and physical activity, had no missing values. We used mean values to deal with the missing values of the two variables. All p-values presented in this study were two-sided and deemed statistically significant at a significance level of p < 0.05. The statistical analyses were conducted using SAS (version 9.4; SAS, Cary, NC, USA). The machine learning algorithms were implemented using R (version 4.3.0) and Python (version 3.10.11).

## 3. Results

### 3.1. Baseline characteristics

The study initially included a total of 56 predictors. After excluding variables that exhibited a high correlation with another one, 48 predictors were finally included in this analysis (Supplemental Figures 1 and 2).

In Table 1, data (n = 2084) were divided into the IR group (HOMA-IR > 2.73, n = 848) and the non-IR group (HOMA-IR ≤ 2.73, n = 1236). The IR group exhibited a higher likelihood of including Black or Hispanic women, possessing a lower family monthly poverty level index, having a higher BMI, engaging in lower levels of physical activity, and being diagnosed with hypertension. Regarding the laboratory data, the IR group had significantly higher mean values of glucose, HDL-C, triglycerides, glycohemoglobin, and total cholesterol than the non-IR group.

The mean values of energy, sugar, cholesterol, saturated fatty acid, alcohol, sodium, and phosphorus intake were significantly different between the IR and non-IR groups. The two groups had no significant differences in the mean values of other daily nutrients.

Women in the IR group had a lower mean age at menarche and more pregnancies than those in the non-IR group. A higher proportion of women in the IR group had histories of hysterectomy or bilateral oophorectomy. However, no significant differences were observed in the number of abortions/miscarriages/stillbirths, oral contraceptive use, and hormone therapy treatment between the two groups.

**Table 1.** Baseline characteristics of 2084 middle-aged nondiabetic women by HOMA-IR.

**Characteristics**	**HOMA-IR > 2.73** IR group (n = 848)	**HOMA-IR ≤ 2.73** Non-IR group (n = 1236)	**p-value**
**Demographic and behavioral variables**			
Age (years)	54.4 (54.0–54.8)	54.1 (53.7–54.4)	0.1723
Race*			<0.0001
Non-Hispanic White	276 (66.6)	521 (74.9)	
Non-Hispanic Black	207 (12.1)	252 (9.0)	
Hispanic	283 (14.7)	302 (8.8)	
Others	82 (6.5)	161 (7.4)	
Education*			0.0985
High school or below	389 (40.3)	517 (34.4)	
College or above	459 (59.7)	719 (65.6)	
Marital status*			0.1553
Married/living with a partner	512 (65.0)	753 (70.0)	
Widowed/divorced/separated	239 (25.4)	353 (22.7)	
Never married	97 (9.6)	130 (7.3)	
Family monthly poverty level index	2.6 (2.5–2.7)	2.8 (2.7–2.9)	0.0066
Smoking*			0.3337
Current	391 (42.9)	576 (47.6)	
Former	185 (25.5)	266 (23.7)	
Never	272 (31.5)	394 (28.7)	
Family history of diabetes*	383 (43.2)	497 (37.8)	0.1260
BMI, kg/m^2^	33.3 (32.9–33.8)	26.8 (26.5–27.1)	<0.0001
Physical activity	14.1 (12.2–16.0)	18.1 (16.3–19.8)	0.0029
Hypertension*	470 (54.4)	480 (33.4)	<0.0001
Systolic BP (mmHg)	126 (125–127)	122 (121–123)	<0.0001
Diastolic BP (mmHg)	72.6 (71.9–73.3)	71.2 (70.7–71.8)	0.0032
**Laboratory variables**			
Glucose (mg/dL)	104.3 (103.7–104.9)	96.1 (95.7–96.6)	<0.0001
HDL-c (mg/dL)	53.4 (52.4–54.3)	64.1 (63.1–65.0)	<0.0001
Triglycerides (mg/dL)	135 (130–140)	105 (100–110)	<0.0001
Glycohemoglobin (%)	5.70 (5.68–5.73)	5.48 (5.47–5.50)	<0.0001
Total cholesterol (mg/dL)	205 (203–208)	209 (207–211)	0.0413
eGFR(mL/min/1.73m^2^)	90.6 (89.4–91.8)	90.8 (89.8–91.7)	0.8456
**Daily intake of nutrients**			
Energy intake (kcal/kg)	22.7 (22.0–23.3)	26.7 (26.0–27.4)	<0.0001
Protein intake ratio (%)	0.16 (0.15–0.16)	0.16 (0.15–0.16)	0.3799
Carbohydrate intake ratio (%)	0.50 (0.49–0.51)	0.49 (0.48–0.50)	0.0741
Total fat intake ratio (%)	0.34 (0.34-0.35)	0.34 (0.33–0.34)	0.1623
Total sugars (gm)	106.9 (102.0–111.9)	96.3 (92.9–99.7)	0.0003
Dietary fiber (gm)	15.9 (15.3–16.6)	16.1 (15.6–16.7)	0.7188
Cholesterol intake (mg)	276 (262–290)	249 (238–261)	0.0029
**Daily intake of nutrients**			
Saturated fatty acid (gm)	23.7 (22.8–24.6)	22.0 (21.2–22.7)	0.0032
Alcohol (gm)	5.4 (4.2–6.6)	8.9 (7.7–10.1)	<0.0001
Vitamin C (mg)	77.1 (71.0–83.2)	81.9 (77.1–86.7)	0.2209
Vitamin D (mcg)	4.4 (4.0–4.8)	4.2 (3.9–4.6)	0.4684
Vitamin B6 (mg)	1.74 (1.67–1.82)	1.77 (1.71–1.84)	0.5562
Vitamin B12 (mcg)	4.2 (4.0–4.5)	4.3 (4.0–4.7)	0.5827
Iron (mg)	12.9 (12.5–13.4)	12.9 (12.5–13.3)	0.8268
Calcium (mg)	849 (816–881)	814 (788–841)	0.1116
Zinc (mg)	9.7 (9.4–10.0)	9.6 (9.2–10.1)	0.8370
Sodium (mg)	3117 (3025–3210)	2971 (2888–3054)	0.0229
Phosphorus (mg)	1224 (1188–1260)	1176 (1147–1206)	0.0434
Copper (mg)	1.15 (1.11–1.19)	1.21 (1.15–1.26)	0.1176
Selenium (mcg)	101.9 (98.5–105.3)	99.1 (95.8–102.5)	0.2761
Potassium (mg)	2427 (2356–2498)	2449 (2389–2509)	0.6365
Theobromine (mg)	30.5 (26.0–35.1)	34.9 (31.1–38.8)	0.1504
Caffeine (mg)	160 (145–175)	162 (151–173)	0.8144
**Reproductive health**			
Age at menarche (years)	12.5 (12.1–12.9)	12.9 (12.7–13.1)	0.0437
Number of pregnancies	2.7 (2.5–2.8)	2.5 (2.3–2.6)	0.0211
Number of abortions/miscarriage/stillbirths	0.71 (0.64–0.79)	0.66 (0.60–0.72)	0.2561
Birth control pills*	531 (64.1)	766 (67.6)	0.1872
Hysterectomy*	215 (25.7)	218 (17.1)	0.0286
Bilateral oophorectomy*	118 (14.3)	102 (8.1)	0.0453
Female hormones*	161 (18.9)	229 (21.6)	0.3432

Note: *represents frequency (weighted proportion, %); other variables were characterized by weighted mean (95% confidence interval). The t-test or the Rao-Scott chi-square test determined the p-value to compare the two IR groups.

### 3.2. Comparing IR prediction models with 48 predictors

We randomly assigned 1667 of 2084 nondiabetic women to the training dataset and 417 to the testing dataset. Table 2 summarizes the performance metrics results of training and testing datasets for XGBoosting, RF, GBM, and DT with 48 predictors. In the training dataset, the AUC of ROC for all models exceeded 0.85, with the maximum AUC value of 0.93 achieved by the XGBoosting model. The XGBoosting algorithm also exhibited superior performance in terms of accuracy (0.86), specificity (0.80), PPV (0.87), NPV (0.85), and F1 score (0.88), followed by the RF, GBM, and DT models.

**Table 2.** The performance metrics of different models with 48 predictors.

	**XGBoost**	**RF**	**GBM**	**DT**
**Training dataset**
AUC of ROC	0.93	0.91	0.90	0.87
Accuracy	0.86	0.82	0.82	0.79
Sensitivity	0.90	0.91	0.87	0.87
Specificity	0.80	0.68	0.75	0.68
PPV	0.87	0.80	0.84	0.80
NPV	0.85	0.84	0.80	0.79
F1 score	0.88	0.85	0.85	0.83
**Testing dataset**				
AUC of ROC	0.86	0.85	0.85	0.80
Accuracy	0.79	0.77	0.78	0.73
Sensitivity	0.86	0.90	0.85	0.84
Specificity	0.69	0.58	0.68	0.56
PPV	0.80	0.76	0.80	0.74
NPV	0.77	0.80	0.76	0.71
F1 score	0.83	0.82	0.82	0.79

Note: XGBoost = Extreme gradient boosting, RF = Random forest, GBM = Gradient boosting machine, DT = Decision tree, AUC of ROC = Area under receiver operating characteristic curve, PPV = Positive predictive value, NPV = Negative predictive value.

**Figure 1.**
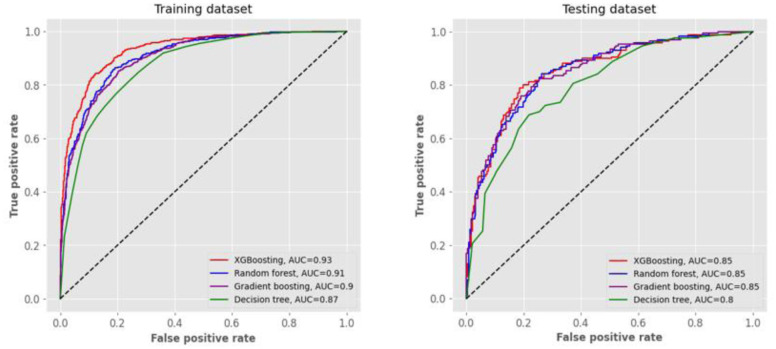
ROC curves for predicting insulin resistance from four different models with 48 predictors. In the training and testing datasets with 48 predictors, the area under curve (AUC) of receiver operating characteristic (ROC) for all four models was higher in XGBoosting, followed by random forest, gradient boosting, and decision tree.

In the testing dataset, all AUCs of ROC were equal to or greater than 0.80, the maximum being 0.86 for XGBoosting. In addition, XGBoosting possessed greater accuracy (0.79), specificity (0.69), PPV (0.80), and F1 score (0.83). The RF, GBM, and DT models had the subsequent performance metrics. Figure 1 also illustrates the ROC curves for predicting IR from the four models with 48 predictors.

### 3.3. Relative importance of 48 predictors in the XGBoosting model

**Figure 2.**
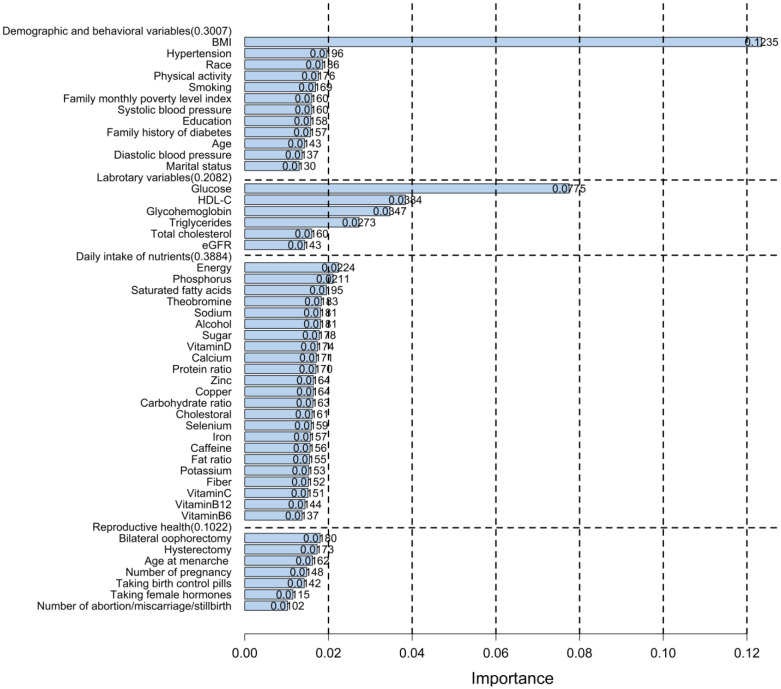
Feature importance of XGBoosting model with 48 predictors. In the XGBoosting model with 48 predictors, the relative importance values of demographic and behavioral factors, laboratory variables, daily intake of nutrients, and reproductive health variables were 0.3007, 0.2082, 0.3884, and 0.1022, respectively.

Figure 2 depicts the relatively important features of the XGBoosting model, with relative importance values of 0.3007, 0.2082, 0.3884, and 0.1022 for demographic and behavioral factors, laboratory variables, daily intake of nutrients, and reproductive health variables, respectively. Among the 48 variables, BMI (0.1235) had the greatest influence on IR, followed by glucose (0.0775), HDL-C (0.0384), glycohemoglobin (0.0347), and triglycerides (0.0273).

### 3.4. Comparing IR prediction models with five predictors

**Figure 3.**
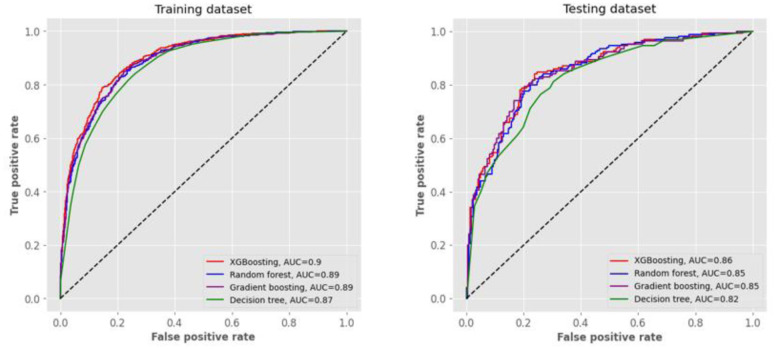
ROC curves for predicting insulin resistance from four models with the top five predictors. In the training and testing datasets with the top five predictors, the area under curve (AUC) of receiver operating characteristic (ROC) for all four models decreased from XGBoosting to random forest, gradient boosting, and decision tree, in descending order.

Based on the above feature importance analysis of the 48 predictors, we attempted to use the top five predictors to develop models. Table 3 presents the performance metrics for the four machine learning algorithms in the training and testing datasets with five predictors. With the top five predictors, the AUC of ROC for all four models stayed at or above 0.87 in the training dataset. The XGBoosting model got a relatively high AUC value of 0.90. In terms of accuracy (0.82), sensitivity (0.87), specificity (0.75), PPV (0.83), NPV (0.79), and F1 score (0.85), the XGBoosting algorithm outperformed the other three algorithms. In the testing dataset, all AUCs of ROC were equal to or greater than 0.83, with the AUC of XGBoosting being the greatest at 0.86. Figure 3 illustrates the ROC curves for predicting IR from the four models with the five predictors. Similar results are shown by the sensitivity analysis for the models' performance metrics using the first five predictors without using the synthetic minority oversampling technique (Supplemental Table 1).

**Table 3.** Performance metrics of different models with the first five predictors.

	**XGBoost**	**RF**	**GBM**	**DT**
**Training dataset**
AUC of ROC	0.90	0.89	0.88	0.87
Accuracy	0.82	0.81	0.79	0.80
Sensitivity	0.87	0.86	0.86	0.85
Specificity	0.75	0.73	0.70	0.72
PPV	0.83	0.82	0.81	0.82
NPV	0.79	0.78	0.77	0.76
F1-score	0.85	0.84	0.83	0.83
**Testing dataset**				
AUC of ROC	0.86	0.85	0.85	0.83
Accuracy	0.78	0.76	0.77	0.76
Sensitivity	0.85	0.84	0.86	0.83
Specificity	0.68	0.66	0.65	0.65
PPV	0.79	0.78	0.78	0.77
NPV	0.75	0.74	0.76	0.73
F1-score	0.82	0.81	0.82	0.80

Note: XGBoost = Extreme gradient boosting, RF = Random forest, GBM = Gradient boosting machine, DT = Decision tree, AUC of ROC = Area under receiver operating characteristic curve, PPV = Positive predictive value, NPV = Negative predictive value.

### 3.5. Relative importance of the five predictors in four models

**Figure 4.**
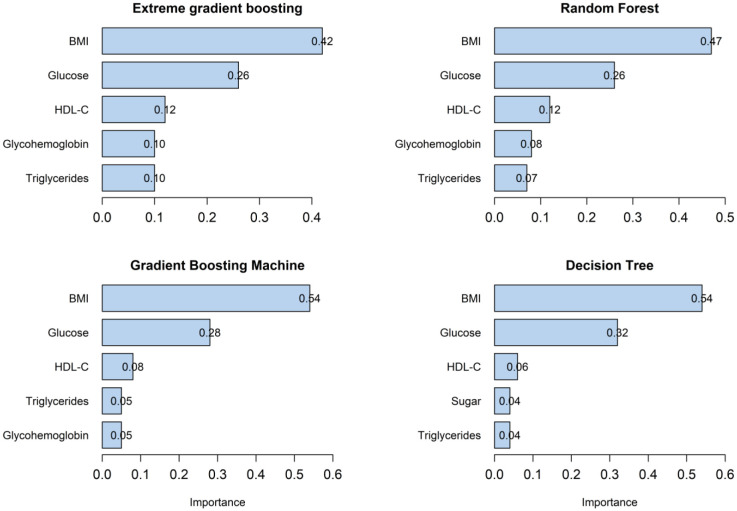
Feature importance of four machine learning models with five predictors. The top five predictors of the XGBoosting, RF, and GBM models were all BMI, glucose, HDL-C, glycohemoglobin, and triglycerides, whereas one of the DT model's predictors was daily sugar intake instead of glycohemoglobin. All four models identified BMI, glucose, and HDL-C as the top three predictors.

The top five predictors in the XGBoosting, RF, and GBM models were BMI, glucose, HDL-C, glycohemoglobin, and triglycerides. However, the DT model included the variable of daily sugar intake instead of glycohemoglobin. Of the five predictors, BMI, glucose, and HDL-C were the top three predictors in all four models. BMI's relative importance was 0.42, 0.47, 0.54, and 0.54 in the XGBoosting, RF, GBM, and DT models, respectively. In the four models, the corresponding relative importance of glucose was 0.26, 0.26, 0.28, and 0.32, while the corresponding relative value of HDL-C was 0.12, 0.12, 0.08, and 0.06 (Figure 4). The sensitivity analysis for the models' feature importance with the first five predictors produces similar results without using the synthetic minority oversampling technique (Supplemental Figure 3).

### 3.6. SHAP value of the five predictors in the XGBoosting model

**Figure 5.**
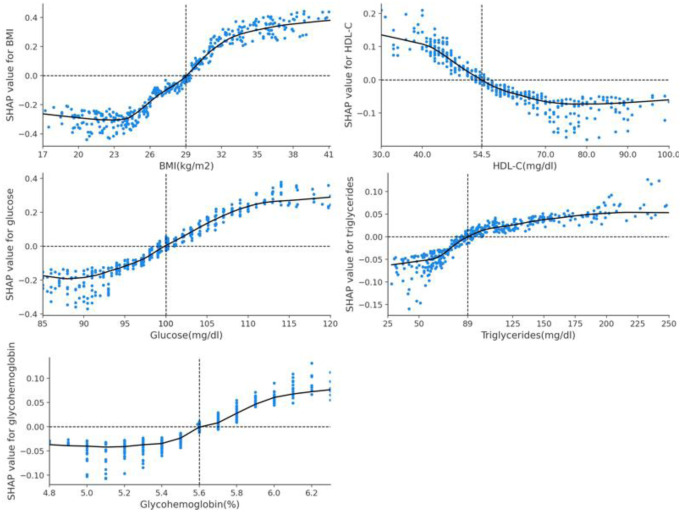
The dependence plot of the five predictors in the XGBoosting model. When the SHAP value is equal to 0, it signifies that the corresponding feature's value does not exert a statistically significant influence on the predictive outcome of the model. The cutoff values for BMI, glucose, HDL-C, triglycerides, and glycohemoglobin are 29 kg/m^2^, 100 mg/dL, 54.5 mg/dL, 89 mg/dL, and 5.6%, respectively.

Figure 5 indicates the relationship between the XGBoosting model's five predictors and their SHAP values. The SHAP values of BMI, glucose, glycohemoglobin, and triglycerides increase as their levels rise. However, as HDL-C increases, its SHAP value decreases. The threshold values for predicting IR were identified to be 29 kg/m^2^, 100 mg/dL, 54.5 mg/dL, 89 mg/dL, and 5.6% for BMI, glucose, HDL-C, triglycerides, and glycohemoglobin, respectively.

**Figure 6.**
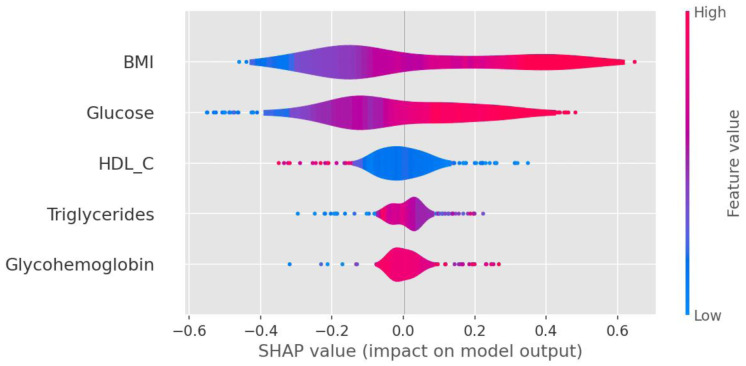
SHAP value of the five predictors in the XGBoosting model. The SHAP values for the XGBoosting model indicate a positive or negative relationship with IR. Specifically, BMI has a strong positive effect on IR. Glucose, triglycerides, and glycohemoglobin all have moderately positive impacts on IR, whereas HDL-C has a medium negative impact.

In Figure 6, the SHAP values for the XGBoosting algorithm reveal the associations between the five predictors and IR, including BMI (strongly positive impact on IR prediction), fasting glucose (strongly positive), HDL-C (medium negative), triglycerides (medium positive), and glycohemoglobin (medium positive). Additionally, the SHAP decision plot further enhances the visualization of the importance and direction of these predictors' contribution, as depicted in Supplemental Figure 4.

## 4. Discussion

We found that the XGBoosting model was the best of the four machine learning algorithms for predicting IR in middle-aged nondiabetic women. The AUC of the ROC curve was 0.90 in the training dataset and 0.86 in the testing dataset using the five predictors of BMI, glucose, HDL-C, glycohemoglobin, and triglycerides. Using the SHAP framework, we also determined the threshold values of the five predictors to predict IR.

The findings of our investigation align with those of prior studies [12]–[14], demonstrating that the XGBoosting algorithm was the optimal model for IR prediction. A study based on the NHANES from 1999 to 2012 reported that the XGBoosting model had a higher AUC of ROC than other machine learning algorithms (RF, logistic regression, and deep neural networks) for predicting IR in 1229 adults with chronic kidney disease [13]. Similarly, another Chinese study recognized XGBoosting as the optimal model with a relatively high AUC value (0.85) out of five machine-learning techniques for predicting IR in 503 children aged 6–12 [14].

XGBoosting is frequently recognized as the optimal model for predicting health conditions due to its ability to capture complex, non-linear relationships between features and the presence of a disease. This makes it well-suited for modeling intricate biological and clinical interactions [39]. Moreover, XGBoosting performs outstanding tasks requiring high accuracy [40]. It provides a range of hyperparameters that can be adjusted to suit the individual attributes of disease datasets. By adjusting the parameters, researchers can refine the model to improve its predictive performance [41].

Previous studies used different numbers of top features, ranging from 5 to 20, to predict diseases of interest [12],[35]. However, we observed that when employing the top five predictors to discriminate IR, the models' performance metrics did not significantly decline compared to the 48 predictors. As an illustration, the AUC for the XGBoosting model in the training dataset decreased from 0.93 to 0.90 when the number of predictors was reduced from 48 to 5.

In addition, the three models XGBoosting, RF, and GBM all identified BMI, glucose, HDL-C, glycohemoglobin, and triglycerides as the top five predictors, and all four models consistently recognized BMI, glucose, and HDL-C as the three most influential predictors. These results exhibited both congruence and divergence in comparison to prior research findings. Multiple prior research has demonstrated significant associations of BMI, glucose, HDL-C, and triglyceride with IR and their potential utility in the IR prediction models [42],[43]. Notably, some studies reported the critical role of blood pressure in predicting IR [44],[45], but neither diastolic nor systolic blood pressure appeared in the first five predictors of our four models. Based on the results depicted in Figure 2, hypertension ranked eighth among the 48 predictors in terms of significance in predicting IR, whereas diastolic and systolic blood ranked outside of the top twenty.

The top three features of the five predictors explained approximately 80% in XGBoosting, 85% in RF, 90% in GBM, and 92% in DT, implying substantial effects of BMI, glucose, and HDL-C on IR (Figure 4). BMI scored unexpectedly highly, despite not being one of the parameters used in calculating HOMA-IR. In alignment with the present study, prior research has documented a robust association between BMI and IR or metabolic syndrome [46],[47]. As BMI increases, the body accumulates more fat, particularly in the abdominal region, which increases the likelihood of insulin resistance [48]. Besides, high BMI may induce a state of chronic low-grade inflammation. Inflammatory signals emitted by adipose tissue can interfere with insulin signaling, decreasing insulin sensitivity in cells [49]. Additionally, high BMI may disrupt the balance of adipokines, which can contribute to IR [50].

The dependent plot shows that the threshold values for predicting IR in the XGBoosting model were 29 kg/m^2^, 100 mg/dL, 54.5 mg/dL, 89 mg/dL, and 5.6% for BMI, glucose, HDL-C, triglycerides, and glycohemoglobin, respectively. These findings are both consistent with and distinct from those of previous research. It is well-known that a glucose level of 100 mg/dL serves as a diagnostic criterion for metabolic syndrome [51], while glucose levels ranging from 100 to 125 mg/dL and/or glycohemoglobin levels of 5.7%–6.4% can be utilized for the diagnosis of pre-diabetes [52]. However, the BMI threshold for IR can vary depending on the study and the population under consideration. A study indicated that a BMI ≥ 25 kg/m^2^ is a risk factor for IR [53], whereas another study found that a BMI ≥ 27 kg/m^2^ is optimal for identifying metabolic syndrome in adult populations [47]. In addition, a noteworthy disparity is that the cutoff level for triglycerides in diagnosing metabolic syndromes is 150 mg/dL [54]. In comparison, our results present the threshold value of 89 mg/dL for IR. These variations may be attributable to differences in the study design and the characteristics of populations, but additional research is necessary to validate our findings.

The SHAP framework provides additional insight into how individual features contribute to the model's predictions, with BMI (strongly), glucose (strongly), glycohemoglobin (moderately), and triglycerides (moderately) having positive impacts on IR, and HDL-C indicating a medium negative impact. The decision plot depicting correct classification and misclassification provides additional evidence of these predictors' significant influence on IR (Supplemental Figure 4). Furthermore, our predictive model broadly agrees with earlier findings [12],[13],[55]. As per the findings from the National Health and Nutrition Examination Survey (2007–09) conducted in South Korea, the XGBoosting model in 8842 individuals aged 40–74 years old indicated that glucose had robust positive effects on IR, and glycohemoglobin (positive) and HDL-C (negative) had moderate effects on IR [12]. Moreover, these predictors are clinically commonplace and simple to measure, suggesting significant promise for middle-aged women's IR screening and prediction.

Despite the excellent accuracy and precision achieved by the XGBoosting model with the top five predictors in predicting IR, it is imperative to acknowledge that the influence of other factors, such as behavioral, nutritional, and reproductive health variables, cannot be disregarded. For instance, energy intake was not chosen as one of the top five predictors for the prediction models. Still, it had the highest feature importance among the variables of daily nutrient consumption. These characteristics can affect BMI, glucose levels, and other laboratory indicators [56]–[58]. Consequently, these anthropometric and laboratory indicators can be employed more directly to identify IR.

This study's strength was its novelty, specifically the inaugural investigation into utilizing machine learning to develop IR predictive models in middle-aged nondiabetic women. Furthermore, the machine learning models utilized in this work incorporated an extensive range of variables, encompassing demographic factors, behavioral lifestyles, laboratory data, and daily intake of macronutrients and micronutrients and reproductive profiles. Besides, the SHAP framework can better explain the impact of feature importance on IR. The clinical significance of our study was that our machine learning–based predictive model could potentially provide women with warnings using routine clinical measurements.

Some limitations existed in our investigation. The data was from a cross-sectional survey, so the findings cannot be interpreted as a cause-and-effect relationship. Demographic, behavioral lifestyle, and reproductive health variables were acquired by self-reported questionnaires, which could potentially be influenced by recall bias. In addition, the lab test was not administered to all NHANES participants, resulting in a sample size of 2084. Increasing the sample size could improve the model's performance [59]. An increased volume of data may enable the model to identify latent patterns more precisely, thereby enhancing its capacity to extrapolate to unobserved data [60]. The presence of IR was evaluated using HOMA-IR rather than hyperinsulinemic-euglycemic clamps. Nevertheless, we posit that our predictive model in middle-aged nondiabetic women can explain IR based on these characteristics in the current dataset.

## 5. Conclusions

In this study, we used four machine learning algorithms, namely XGBoosting, random forest, gradient boosting, and decision tree, to identify IR in 2084 middle-aged women who do not have diabetes. Our analysis involved 48 variables encompassing demographic and behavioral factors, laboratory variables, daily nutrient intake, and reproductive health variables. The XGBoost algorithm demonstrated a relatively high AUC of ROC, followed by the RF, GBM, and DT models. When modeled with the top five predictors, the XGBoosting model's performance metrics remain optimal, with BMI (strongly positive impact), fasting glucose (strongly positive impact), HDL-C (medium negative impact), triglycerides (medium positive impact), and glycohemoglobin (medium positive impact) being associated with IR.

## Use of AI tools declaration

The authors declare they have not used Artificial Intelligence (AI) tools in the creation of this article.


