# Supplementary material for: Discriminating insulin resistance in middle-aged nondiabetic women using machine learning approaches
Source: AIMS Public Health. 2024 May 9;11(2):667–87. doi: 10.3934/publichealth.2024034 (PMC11252584; doi:10.3934/publichealth.2024034)
Supplement: Supplementary file 1 [file publichealth-11-02-034-s001.pdf]

Public Health

AIMS Public Health, 11(2): 667–687.
DOI: 10.3934/publichealth.2024034
Received: 20 February 2024
Revised: 28 March 2024
Accepted: 08 April 2024
Published: 09 May 2024

https://www.aimspress.com/journal/aimsph

Research article

Discriminating insulin resistance in middle-aged non-diabetic women using machine learning approaches

Zailing Xing*, **Henian Chen and Amy C. Alman**

College of Public Health, University of South Florida, 13201 Bruce B. Downs Blvd, MDC 56, Tampa, FL 33612, USA

* **Correspondence:** Email: xingzailing@gmail.com.

Supplementary

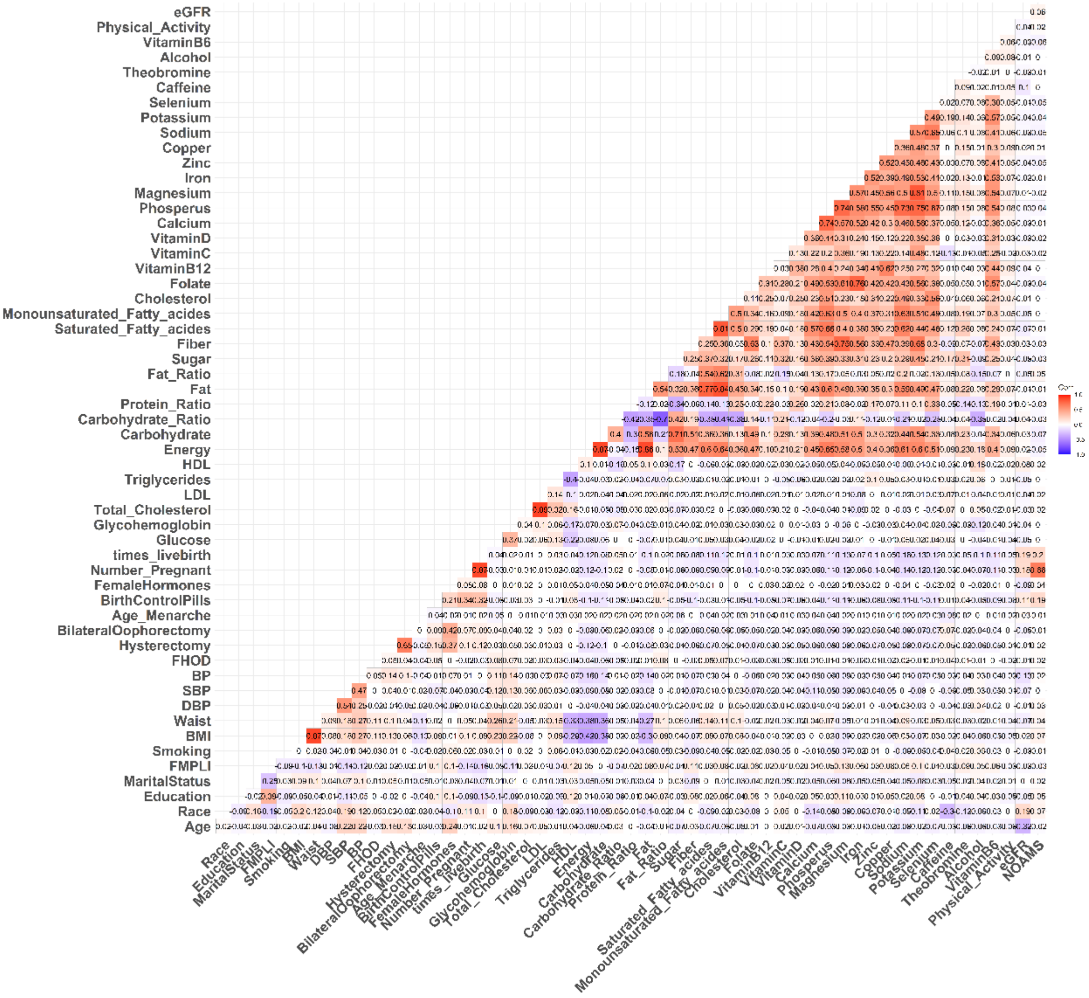

Supplemental Figure 1. Correlation matrix of original 56 predictors in the study.

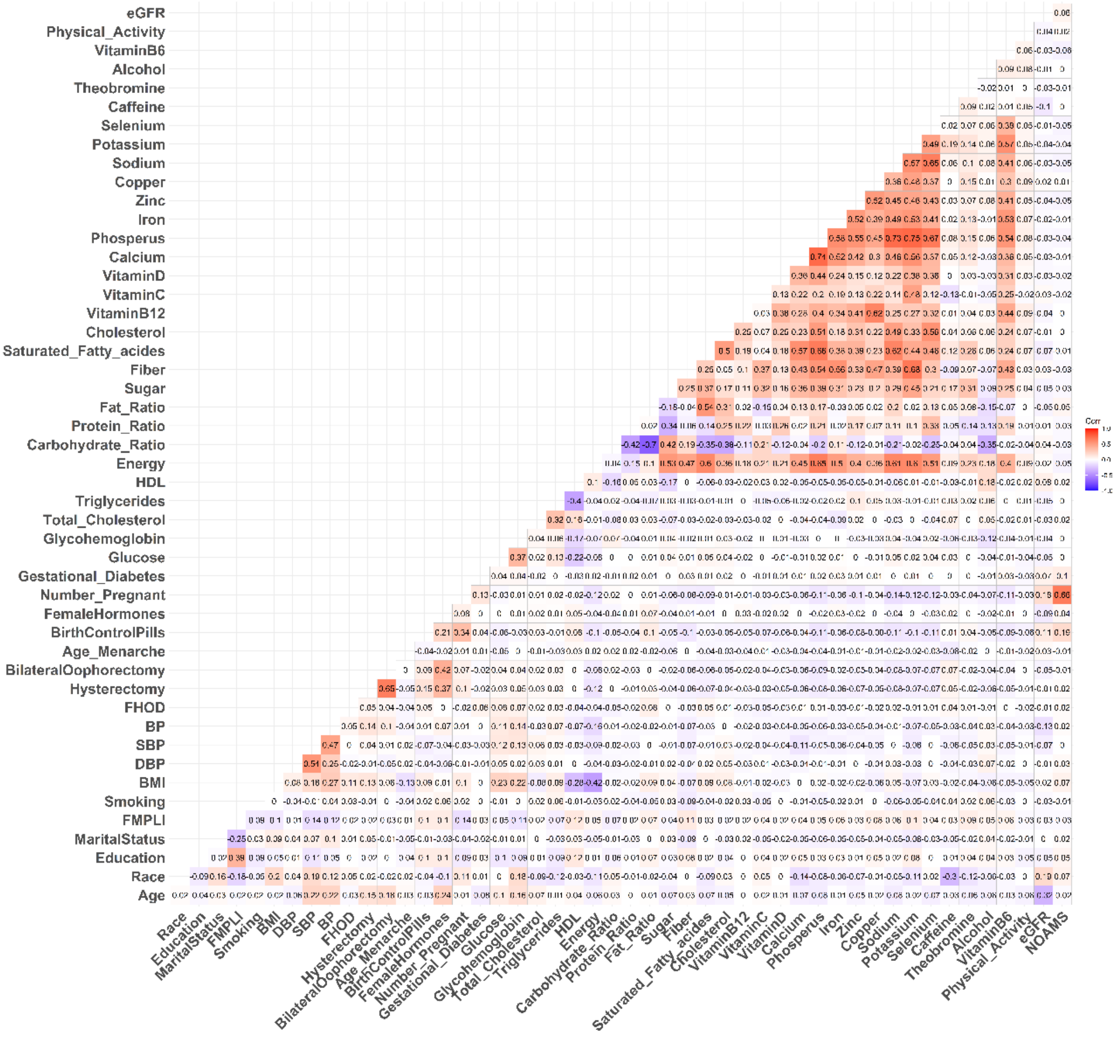

Supplemental Figure 2. Correlation matrix of 48 predictors in the study after removing highly correlated predictors. Note: After excluding variables that displayed a strong correlation with one another (correlation coefficient >0.75), 48 predictors were ultimately incorporated into this study.

Supplemental Table 1. The sensitivity analysis for the performance metrics of different models with the first five predictors without using the synthetic minority over-sampling technique.

	XGBoost	RF	GBM	DT
**Training dataset**				
AUC of ROC	0.90	0.89	0.88	0.87
Accuracy	0.82	0.82	0.80	0.80
Sensitivity	0.86	0.86	0.86	0.87
Specificity	0.76	0.75	0.72	0.69
PPV	0.84	0.84	0.82	0.81
NPV	0.79	0.78	0.78	0.78
F1-score	0.85	0.85	0.84	0.84
**Testing dataset**				
AUC of ROC	0.84	0.85	0.84	0.82
Accuracy	0.79	0.79	0.78	0.77
Sensitivity	0.85	0.83	0.83	0.84
Specificity	0.70	0.72	0.70	0.65
PPV	0.82	0.83	0.82	0.79
NPV	0.74	0.72	0.72	0.71
F1-score	0.83	0.83	0.82	0.82

Note: XGBoost = Extreme gradient boosting, RF = Random forest, GBM = Gradient boosting machine, DT = Decision tree, AUC of ROC = Area under receiver operating characteristic curve, PPV = Positive predictive value, NPV = Negative predictive value.

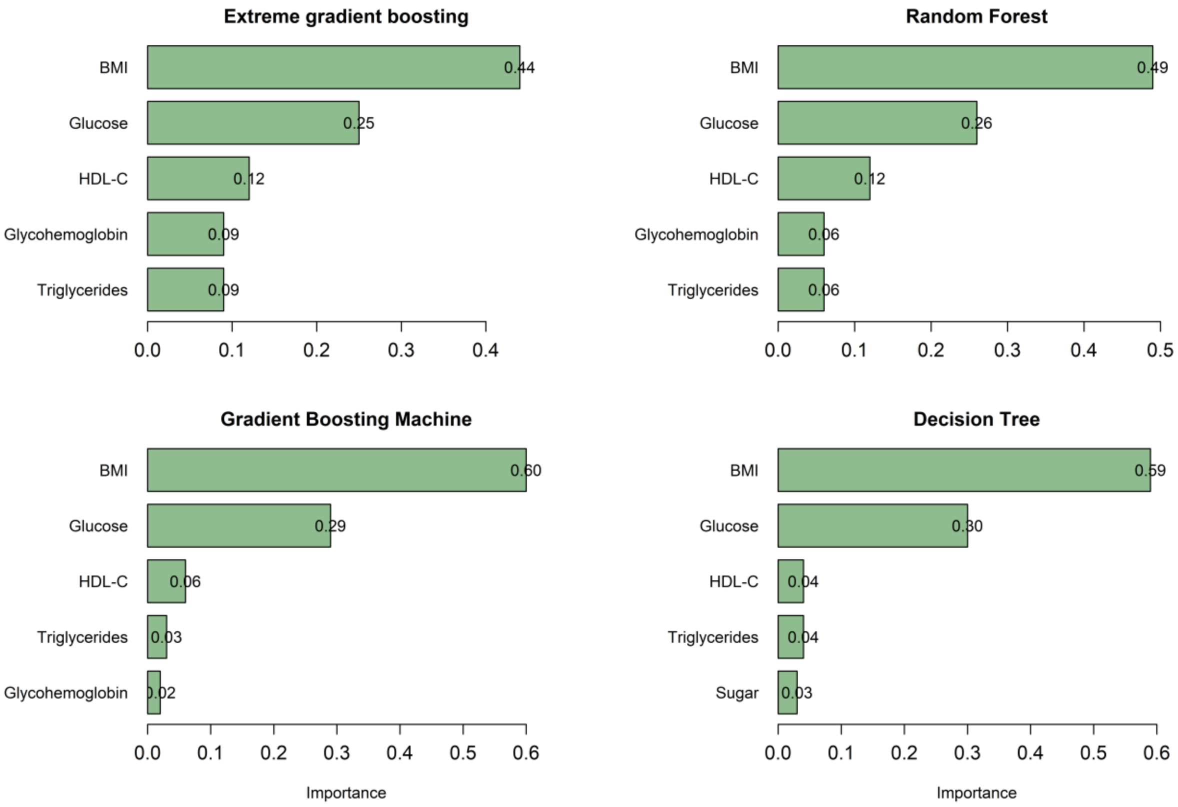

Supplemental Figure 3. The feature importance of four machine learning models with the top five predictors without using the synthetic minority over-sampling technique.

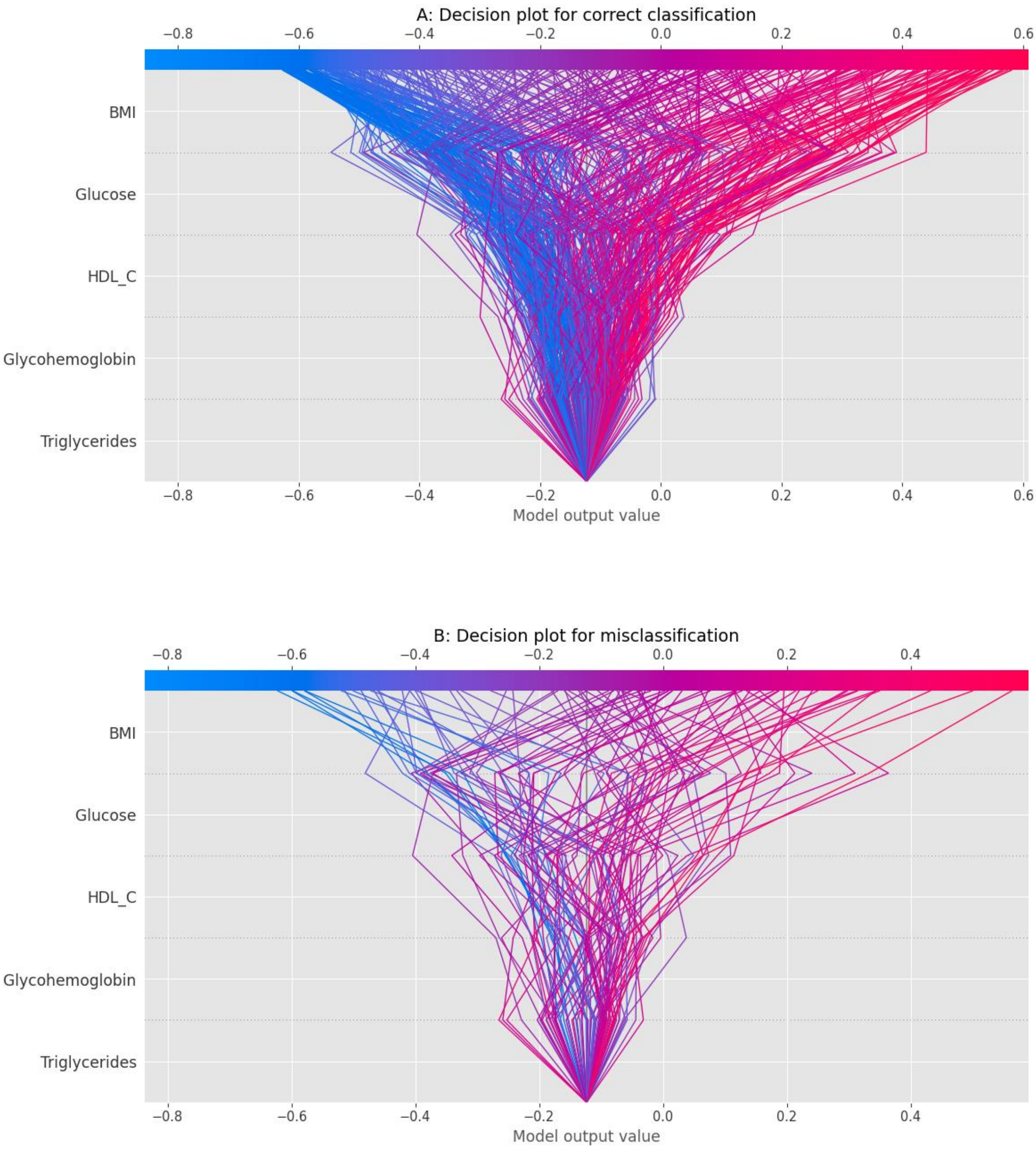

Supplemental Figure 4. SHAP decision plot for correct classification and misclassification in the XGBoosting model. Note: The first figure (A) presents the decision plot for the correct classification, while the second figure (B) illustrates the decision plot for the misclassification. These plots, with the vertical axis representing the model's predictors, provide a visual representation of how the SHAP values build from the base value to the model's ultimate score at the top, enhancing our understanding of the XGBoosting model.

AIMS Press

© 2024 the Author(s), licensee AIMS Press. This is an open access article distributed under the terms of the Creative Commons Attribution License (https://creativecommons.org/licenses/by/4.0)
